# Host-Feeding Patterns of *Culex* Mosquitoes in Relation to Trap Habitat

**DOI:** 10.3201/eid1312.070275

**Published:** 2007-12

**Authors:** Lisa A. Patrican, Laura E. Hackett, James E, Briggs, Jay W. McGowan, Thomas R. Unnasch, Joon-Hak Lee

**Affiliations:** * State of New York Department of Health, Ithaca, New York, USA; † University of Alabama at Birmingham, Birmingham, Alabama; ‡ State of New York Department of Health, Albany, New York, USA; 1 Current affiliation: Cornell University, Ithaca, New York, USA; 2 Current affiliation: State of New York Department of Health, Syracuse, New York, USA; 3 Current affiliation: University of North Texas–Health Science Center, Fort Worth, Texas, USA

**Keywords:** mosquito, Culex, blood meal identification, host feeding patterns, West Nile virus, dispatch

## Abstract

Mosquito feeding patterns identify vertebrate species potentially involved in the amplification of West Nile virus. In New York, northern cardinals (*Cardinalis cardinalis*) were the predominant hosts in most habitats. Crow (*Corvus* sp.) blood meals were most frequently identified from sewage treatment plant and storm water catch basin habitats.

In the northeastern United States, *Culex pipiens* and *Cx. restuans* are the most important vectors of West Nile virus (WNV), according to the frequency of viral detection, vector competence, and their largely ornithophilic feeding habits (*1*–*4*). Mosquito feeding studies suggest that American robins (*Turdus migratorius*) are the preferred avian hosts that support enzootic transmission in the Northeast (*4*,*5*). A shift in hosts from birds to mammals, including humans, as robins begin fall migratory movements is hypothesized to be responsible for the seasonal rise in human WNV cases (*5*). We identified blood meals of *Culex* mosquitoes collected in New York and found feeding patterns unlike those previously reported (*4*,*5*). We suggest caution in applying findings for epidemiologic purposes across different habitats and large geographic areas.

## The Study

We determined host species of *Cx. pipiens* and *Cx. restuans* mosquitoes collected by mosquito surveillance programs in 2001 and 2002 in Nassau (89 identified/100 tested), Orange (66/87), Rockland (83/96), and Westchester (20/20) counties and in 2005 and 2006 in Tompkins County (46/52) (*1*). Dry ice–baited CDC light traps and gravid traps were used to capture host-seeking and ovipositing females, respectively. Seventy percent of mosquitoes were collected from traps located in public places such as parks, preserves, woodlots, cemeteries, and golf courses (hereafter, parks and preserves). The remaining 30% were collected on residential properties, near storm water basins and sewage treatment plants, and at a university composting facility and dairy barn. *Culex* species were identified molecularly with taxon-specific primers (*6*).

Genomic DNA was extracted from each mosquito by using DNAzol-BD (Molecular Research Center, Cincinnati, OH, USA) or DNeasy Blood & Tissue Kits (QIAGEN, Germantown, MD, USA). Blood meals were initially identified by PCR-heteroduplex assays (*7*). We subsequently used DNA sequencing with cytochrome b primers as follows: Cyt F 5′-GCHGAYACHWVHHYHGCHTTYTCHTC-3′ and Cyt H 5′-CCCCTCAGAATGATATTTGTCCTCA-3′, in which W = A or T, H = A, C, or T, Y = C or T, and V = A, C, or G. Cycling conditions were 94°C for 2 min, followed by 55 cycles at 94°C for 45 s, 50°C for 50 s, and 72°C for 1 min with a final extension at 72°C for 7 min. PCR amplifications were conducted by using Taq PCR Core Kits (QIAGEN). Expected 300-bp PCR products were purified with an exonuclease-alkaline phosphate kit (Exo SAP-IT, USB Corporation, Cleveland, OH, USA). Samples were sequenced at the Biotechnology Resource Center (Cornell University, Ithaca, NY, USA) with a 3730 DNA Analyzer (Applied Biosystems, Foster City, CA, USA). Sequences were identified by using BLASTn searches in the GenBank database to compare fragments (*8*).

We identified host species in 183 *Cx. pipiens* and 119 *Cx. restuans* (Appendix Table). *Cx. pipiens* fed on birds (n = 171, 92.9% of *Cx. pipiens* blood meals), mammals (n = 12, 6.5%), and a northern brown snake (*Storeria d. dekayi*) (n = 1, 0.5%). *Cx. restuans* fed exclusively on birds. Avian host species were similar to those previously reported (*3*,*4*), except that northern cardinals (*Cardinalis cardinalis*), not American robins, were the principal hosts throughout the season, and feeding patterns differed somewhat, depending on the habitat of the trap site. Mosquitoes trapped in parks and preserves fed on 32 species of birds. Northern cardinal, gray catbird (*Dumetella carolinensis*), American robin, and blue jay (*Cyanocitta cristata*) accounted for 64% of the identifications. On residential properties, 52% of the blood meals were from cardinals. American robin blood meals accounted for only 12% of the blood meals and were found only in parks, preserves, and residential and storm water catch basins habitats. Crows (*Corvus* spp.) accounted for 26% of the blood meals from storm water catch basins and sewage treatment plant sites but only 2% of the collections from parks and preserves. No crow blood meals were identified from other habitats. American crows (*C. brachyrhynchos*) and fish crows (*C. ossifragus*) are found where crow-fed mosquitoes were collected. Mammalian blood meals were identified in June (human, white-tailed deer, and raccoon), July (deer and Virginia opossum), and August (human, 3 white-tailed deer, and 2 eastern gray squirrels). The percentage of northern cardinal and American robin blood meals was relatively constant throughout the summer (p = 0.261, Fisher exact test) (Table). The proportion of gray catbird–derived blood meals increased somewhat late in the summer (p = 0.668, Fisher exact test).

**Table:** Monthly prevalence of predominant avian hosts of *Culex pipiens* and *Cx. restuans,* New York

Month	Total no. blood meals identified	Northern cardinal				American robin				Gray catbird		
		*Cx. pipiens*	*Cx. restuans*	% of total		*Cx. pipiens*	*Cx. restuans*	% of total		*Cx. pipiens*	*Cx. restuans*	% of total
May	7		4	57.1			1	14.3				
Jun	83	5	13	21.7			11	13.3		1	4	6
Jul	99	22	17	39.4		7	*3*	10.1		3	1	4
Aug	82	21		25.6		11		13.4		14	1	18.3
Sep	18	7		38.9		1		5.6		6	1	38.9
Oct	1	1		100								

The avifauna was not censused in Nassau, Orange, Rockland and Westchester Counties. However, data on breeding bird communities were available for mosquito trap locations, and all species detected in blood meals were known to be present there (*9*,*10*). In Tompkins County, 10-min point-count censuses within a 50-m radius of mosquito traps were conducted 2–3 times each month from June through September 2006, totaling 140 counts. Sites included 8 residential properties, a university composting facility, and a wooded area. Of the 84 avian species recorded, the most frequent were northern cardinal (n = 110), black-capped chickadee (*Poecile atricapillus*) (n = 109), American robin (n = 103), blue jay (n = 102), and American crow (n = 100). Although the relative frequency of northern cardinals and American robins was approximately the same at Tompkins County sites, northern cardinals were 7.7 times more likely than American robins to be selected at those sites.

## Conclusions

We found northern cardinals, rather than American robins, to be the predominant hosts of *Cx. pipiens* and *Cx. restuans* in all habitat types except storm water catch basins, where crows were identified most frequently. Robin-derived blood meals were less common than reported elsewhere (*4*,*5*). No seasonal decline in robin-fed *Cx. pipiens* or shift to other birds or mammals was found. We found that 7% of *Cx. pipiens* fed on mammals, similar to findings of a study in Connecticut (*4*).

The infrequent identification of crow-derived blood meals relative to their local abundance is an enigma (*3*,*4*). However, the spatial and temporal distribution and social behavior of crows have never been considered. Unless mosquito traps are located where crows are present at dusk or sleeping, the probability of collecting a crow-fed mosquito in the area sampled is low. This caveat is particularly relevant for mosquitoes with relatively short flight ranges such as *Cx. pipiens* and *Cx. restuans,* which presumably would not travel far to find suitable hosts.

Why American robins were the predominant hosts found in host-feeding studies in Connecticut, Maryland, and Washington, DC (*4*,*5*) and why northern cardinals were the preferred hosts in our study are not clear. At the Tompkins County, New York, sites, the relative abundance of cardinals and robins was comparable throughout the season. Thus, host abundance does not explain the frequency of cardinal-derived blood meals, at least at those sites. Cardinals and robins are common, share similar habitats, and are capable of amplifying WNV (*11*,*12*). WNV seroprevalence rates in northern cardinals, American robins, and other birds differ across geographic areas from year to year (*13*). Whether host-feeding patterns parallel those findings is not known.

*Cx. pipiens* fed on humans in June and August and on deer throughout the summer. In areas experiencing recurrent human WNV infection, future blood meal analyses should focus on peridomestic populations of *Culex* spp. to better understand their predilection for avian and/or mammalian feeding and the spatial and temporal dynamics of their host-feeding activities.

## Supplementary Material

Appendix Table Feeding patterns of Culex pipiens (PIP) and Cx. restuans (RES) collected in New York State*
