# Supplementary material for: Host-Feeding Patterns of Culex Mosquitoes in Relation to Trap Habitat
Source: Emerg Infect Dis. 2007 Dec;13(12):1921–3. doi: 10.3201/eid1312.070275 (PMC2876745; doi:10.3201/eid1312.070275)
Supplement: Appendix Table — Feeding patterns of Culex pipiens (PIP) and Cx. restuans (RES) collected in New York State* [file 07-0275_appT-s1.pdf]

Appendix Table. Feeding patterns of *Culex pipiens* (PIP) and *Cx. restuans* (RES) collected in New York State*

		Parks, preserves, woodlots, and golf courses†			Residential properties‡			Storm water catch basins§			Sewage treatment plants¶			Compost pile and dairy barn#		
Species	No.	PIP, n = 127	RES, n = 85	% of total, n = 212	PIP, n = 24	RES, n = 9	% of total, n = 33	PIP, n = 18	RES, n = 17	% of total, n = 35	PIP, n = 10	RES, n = 6	% of total, n = 16	PIP, n = 4	RES, n = 2	% of total, n = 6
Birds																
Northern cardinal (*Cardinalis cardinalis*)	90	38	21	27.8	11	6	51.5	4	2	17.1	3	3	37.5		2	33.3
American robin (*Turdus migratorius*)	34	9	14	10.8	3	1	12.1	7		20						
Gray catbird (*Dumetella carolinensis*)	31	23	7	14.2	1		3									
Blue Jay (*Cyanocitta cristata*)	18	11	7	8.5												
Crow (*Corvus sp.*)	17		4	1.9				3	6	25.7	1	3	25			
House sparrow (*Passer domesticus*)	16	8	3	5.2				1		2.9	2		12.5	2		33.3
Common grackle (*Quiscalus quiscula*)	14	9	3	5.7							2		12.5			
Song sparrow (*Melospiza melodia*)	10	2	2	1.9	4	1	15.1							1		16.7
European starling (*Sturnus vulgaris*)	7		5	2.4					2	5.7						
Mourning dove (*Zenaida macroura*)	6	3		1.4				1	2	8.6						
Others	47	16	19	16.5	2	1	9.1	2	5	20	2		12.5			
Mammals																
Humans	2				2		6.1									
Deer (*Odocoileus virginianus*)	5	3		1.4	1		3							1		16.7
Grey squirrel (*Sciurus carolinensis*)	2	2		0.9												
Virginia opossum (*Didelphis virginiana*)	1	1		0.5												
Northern raccoon (*Procyon lotor*)	1	1		0.9												
Northern brown snake (*Storeria d. dekayi*)	1	1		0.9												

*Total no. of trap nights by county ranged from 2,505 to 1,172 (mean (1,735).
†Includes 48 sites in Nassau (15), Rockland (18), Orange (14), Westchester (12), and Tompkins (1) Counties.
‡Includes 8 sites in Tompkins County.
§Includes 14 sites in Nassau County.
¶Includes 8 sites in Orange (4), Nassau (3), and Rockland (1) Counties
#Includes 2 sites in Tompkins County.
